# To Remove Stains from Marble

**Published:** 1914-12

**Authors:** 


**To Remove Stains From Marble:** Soft soap, 4; whiting, 4; dried sodium carbonate 1 part. Water to make a thin paste. Apply to the soiled surface and wash off after 24 hours.

